# Germline Variants in Proto-Oncogenes and Tumor Suppressor Genes in Women with Cervical Cancer

**DOI:** 10.3390/biomedicines12112454

**Published:** 2024-10-25

**Authors:** Ksenia Lenkova, Rita Khusainova, Raushaniya Minyazeva, Aliya Zaripova, Irina Gilyazova, Natalia Mokrysheva, Ildar Minniakhmetov

**Affiliations:** 1 Institute of Biochemistry and Genetics—Subdivision of the Ufa Federal Research Centre of the Russian Academy of Sciences, Prospekt Oktyabrya Street, 71, 450054 Ufa, Russia; khusainova.rita@endocrincentr.ru (R.K.); a.ramilna@bk.ru (A.Z.); gilyasova_irina@mail.ru (I.G.); 2 Endocrinology Research Centre, Dmitry Ulyanov Street, 11, 117292 Moscow, Russia; mokrisheva.natalia@endocrincentr.ru; 3 Internal Medicine Department, Bashkir State Medical University, 450008 Ufa, Russia; dr.gubaydullina@mail.ru; 4 Faculty of Biology, Saint Petersburg State University, Universitetskaya nab. Street, 7/9, 199034 St. Petersburg, Russia

**Keywords:** cancer, oncogenes, tumor suppressor genes, molecular genetic landscape

## Abstract

Background/Objectives: Cervical cancer (CC) remains a significant global health challenge, characterized by genetic heterogeneity and a complex molecular landscape, both of which contribute to its pathogenesis. This study aimed to investigate germline variants in proto-oncogenes and tumor suppressor genes in cervical cancer patients, with the objective of clarifying their potential role in disease development. Methods: We utilized a custom next-generation sequencing (NGS) panel targeting 48 genes implicated in oncogenesis. Germline DNA samples from cervical cancer patients were analyzed in order to identify nucleotide sequence alterations. Variants were classified according to pathogenicity and clinical relevance, based on established guidelines. Results: A total of 148 nucleotide variants were detected within the cohort. Of these, 35 variants (23.6%) were classified as benign. In contrast, 105 variants (70.9%) were identified as variants of uncertain significance (VUSs). Moreover, seven pathogenic or likely pathogenic mutations were discovered, along with the polymorphic variant rs1042522 in the *TP53* gene, which has been associated with an increased risk of cervical cancer. Conclusions: Our findings contribute to expanding our understanding of the molecular genetic landscape of cervical cancer. They emphasize the potential contribution of rare germline mutations to its development and progression. These results highlight the importance of comprehensive genetic screening in order to improve diagnostic and therapeutic approaches for cervical cancer patients.

## 1. Introduction

Cervical cancer ranks 4th in the structure of oncological diseases and cancer mortality among women in 2022, with approximately 660,000 new cases and around 350,000 deaths reported worldwide. A significant trend has been noted in the increasing prevalence of cervical cancer in sub-Saharan Africa, Central America, and Southeast Asia. Of the 350,000 deaths caused by cervical cancer, 94% occurred in low- and middle-income countries [1,2].

Infection caused by human papillomavirus (HPV) remains a major risk factor for developing cervical cancer. Additionally, many other genetic and epigenetic factors are involved in the pathogenesis of cervical cancer [3]. Cervical cancer is predominantly classified as either squamous-cell carcinoma or adenocarcinoma, and its development may be influenced by potentially oncogenic germline mutations. Notably, squamous-cell carcinoma and adenocarcinoma of the cervix exhibit different molecular profiles [4]. Most known cancer predisposition genes are involved in maintaining genomic integrity, protecting DNA from mutations that could ultimately lead to malignant tumors. Current data suggest that, besides increasing cancer risk, germline variants also affect tumor progression, shaping the landscape of somatic alterations in cancer [5,6].

Recently, several studies have been conducted to identify germline variants in patients with cervical cancer, aiming to discover predisposition genes or factors influencing disease progression. A meta-analysis of GWAS identified several candidate genes most likely associated with cervical cancer predisposition, including *PAX8/PAX8-AS1*, *LINC00339*, *CDC42*, *CLPTM1L*, *HLA-DRB1*, and *GSDMB* [7]. Despite numerous sequencing studies, the genetic characteristics of cervical cancer are still not fully understood, particularly in populations not included in GWAS. Lin Qiu and colleagues conducted a parallel study on the Chinese population using a multigene NGS panel targeting 571 cancer-related genes. In this study, a total of 810 somatic variants, 2730 germline mutations, and 701 copy number variations (CNVs) were detected. The genes *FAT1*, *HLA-B*, *PIK3CA*, *MTOR*, *KMT2D*, and *ZFHX3* were the most frequently mutated. Additionally, loci in the genes *PIK3CA*, *BRCA1*, *BRCA2*, *ATM*, and *TP53* exhibited a higher frequency of CNVs. This study demonstrated that genetic variations not only influence the predisposition to cervical cancer but also affect the resistance of cervical cancer to radiotherapy. However, additional studies involving larger patient cohorts are required to confirm these findings [8]. A study by Hao Wen and colleagues on the prevalence of pathogenic and potentially pathogenic germline variants in 62 cancer predisposition genes in the Chinese population demonstrated that the prevalence of pathogenic and potentially pathogenic variants was 6.4% (23 out of 358) for cervical cancer [9].

The aim of this study is to identify germline mutations in proto-oncogenes and tumor suppressor genes that play key roles in the molecular pathogenesis of tumors. By considering clinical data from patients, we aim to assess the significance of these mutations in forming genetic predispositions to cervical cancer and their impact on disease progression.

## 2. Materials and Methods

### 2.1. Patient Samples

The study sample consisted of 108 unrelated women with a clinical diagnosis of cervical cancer residing in the Republic of Bashkortostan (a region at the crossroads of Eastern Europe and Asia). The examination, final diagnosis, and treatment of the patients were carried out at the Republican Clinical Oncology Dispensary of the Ministry of Health of the Republic of Bashkortostan. The sample includes women born between 1951 and 1994, with an average age of 50 years. The HPV status of most patients is not formally documented, as it is generally presumed positive and does not influence the treatment approach. The choice of treatment method for cervical cancer is determined individually and depends on the extent of the tumor process and the severity of concomitant somatic pathology. The primary treatment strategy for this cohort of patients at stages IA1-IA2 is surgical intervention, while at stages IB and IIA, surgical treatment or radiation therapy/chemoradiotherapy may be applied. At stages IIB–IVA, the standard treatment is chemoradiotherapy under a radical protocol—combined radiation therapy (external beam radiation therapy + brachytherapy) with weekly administration of cisplatin at 40 mg/m^2^ for 5–6 cycles during external beam radiation therapy [10,11]. The majority of women in the study sample had squamous-cell carcinoma (89.1%), while adenocarcinomas, including mucinous tumors, accounted for 7.92%, adenosquamous cervical cancer for 0.99%, aplastic cervical cancer for 0.99%, and leiomyosarcoma for 0.99%, with varying degrees of differentiation and stages of disease from in situ to metastatic. The women in the sample represented the three most common ethnic groups in the Republic of Bashkortostan: Russians, Tatars, and Bashkirs. The comparison group for the *TP53* gene polymorphism rs1042522 and the SNP rs17879961 in the *CHEK2* gene consisted of DNA from 51 women who had cleared HPV, indicating that the virus did not persist or lead to cervical cancer. For additional studies aimed at determining microsatellite instability in the somatic DNA of patients with potentially pathogenic variants in the dMMR gene system in germline DNA, histological blocks with formalin-fixed and paraffin-embedded malignant tumor tissue were used.

### 2.2. NGS Analysis

Genomic DNA was extracted from peripheral blood lymphocytes using the phenol-chloroform extraction method as described by Mathew (1984). The concentration and purity of the extracted DNA were assessed using a Qubit 3.0 fluorometer (Thermo Fisher Scientific, Singapore) and 1.5% agarose gel electrophoresis. A custom NGS panel was used in the study to analyze 48 genes, 27 of which function as proto-oncogenes and tumor suppressors (Table 1). The panel was primarily designed to study exons. However, due to the random fragmentation of DNA during the initial pre-processing step, the resulting fragments may include both targeted regions and adjacent intronic regions. Consequently, variants were also identified in the intronic regions of the genes. The 37th genomic assembly (GRCh37) was used to assign and search for genomic coordinates in this study. Sample libraries for NGS analysis were prepared using the KAPA Hyper Cap Workflow v3.0 reagent kit (Roche Diagnostics, Mannheim, Germany). Sample libraries for NGS analysis were prepared using the KAPA Hyper Plus Kit for enzymatic fragmentation of genomic DNA (gDNA) (Roche Diagnostics). In the amplification and library purification step, KAPA UDI Primer Mixes (Roche Diagnostics) and KAPA Hyper Pure Beads (Roche Diagnostics) were used. In addition, the KAPA Hyper Choice reagent kit (Roche Diagnostics) was used to capture target regions of human gDNA up to 200 Mb in size. KAPA Target Enrichment Probes (Roche Diagnostics) were used to enrich target libraries. All steps of the library preparation, including multiplexing, hybridization, and enrichment, were carried out according to the manufacturer’s instructions. NGS sequencing was performed using the MiSeq Series next-generation sequencer with the MiSeq Reagent Kit v2 (300cycles) (Illumina, San Diego, CA, USA).

### 2.3. Data Processing and Additional Studies

Secondary analysis of the obtained FASTQ files was performed separately for each sample using a BED file containing genomic coordinates and a Manifest file with a similar coordinate format, within the Illumina BaseSpace cloud data analysis service, specifically using the “BWA Enrichment” application (Illumina, San Diego, CA, USA). As a result, we obtained VCF files containing information about sequence variations in the studied DNA compared to the reference genome, and we analyzed the samples using the Variant Interpreter database (Illumina, San Diego, CA, USA). Bioinformatic processing protocols were developed based on the recommendations provided by Illumina. The company’s platform was used for primary data processing, followed by annotation of the identified variants. These annotations included all known transcripts of each gene from the RefSeq database, using computational algorithms to predict the pathogenicity of the variants. The predictions were made in accordance with the guidelines of the American College of Medical Genetics and Genomics (ACMG) [12,13,14]. The identified nucleotide sequence variations were classified into six groups: benign (B), likely benign (LB), pathogenic (P), likely pathogenic (LP), variants of uncertain significance (VUSs), and variants absent from the utilized database. Subsequent verification and analysis of the clinical significance of the detected mutations were conducted using the “ClinVar”, “BSKN”, and “OncoBRCA” databases, taking into account clinical information. Data on the prevalence of variants and pathogenicity prediction program calculations (SIFT, PolyPhen-2, CADD, REVEL, MetaLR, MutationAssessor) were obtained using the online resources “Ensembl” (Ensembl release 113) and “GnomAD” (v4.0.0). Functional analysis of the protein products was performed using resources for building protein spatial models and determining changes within them: “SWISS-MODEL”, “DynaMut2”, and “UniProt”. Statistical data processing was carried out using the Plink web resource for whole-genome association analysis (https://zzz.bwh.harvard.edu/plink/data.shtml) (accessed on 15 May 2024), utilizing Microsoft Windows tools, including Excel (Microsoft Office 2010, Redmond, WA, USA) and Notepad (Microsoft Office 2010, Redmond, WA, USA). The significance threshold was set at *p* < 0.05. Real-time polymerase chain reaction (RT-PCR) for rs1042522 was conducted using TaqMan technology with the “Syntol” reagent kit (Russia) and the “CFX96” detecting amplifier (BioRad, Hercules, CA, USA) according to the manufacturer’s instructions. RT-PCR for rs17879961 was conducted using polymorphism detection reagents from “DNA-Synthesis” (DNA-Synthesis, Moscow, Russia) with the “DTprime” real-time PCR detection system (DNA Technology, Protvino, Russia), following the manufacturer’s protocol. The rs17879961 variant was selected for additional study due to its frequency of 2.7% among cervical cancer patients, compared to 0.92% for other pathogenic and likely pathogenic variants. Immunohistochemical analysis of microsatellite instability, performed using the “VENTANA MMR” panel, and molecular–genetic analysis of microsatellite instability (MSI) were conducted on a commercial basis. A 2–4 μm thick slice is made from formalin-fixed, paraffin-embedded (FFPE) tumor tissue and an immunohistochemical reaction is performed using the VentanaBenchMark ULTRA immunohistostainer (Roche Diagnostics, Mannheim, Germany) and antibodies to proteins involved in the mismatch repair (MMR) system. The assay requires the OptiView DAB IHC Detection Kit (Roche Diagnostics, Mannheim, Germany) and the monoclonal antibodies MLH1, MSH2, MSH6, and PMS2 (Roche Diagnostics, Mannheim, Germany), with mandatory use of the OptiView Amplification Kit signal amplifier for the latter antibody.

## 3. Results

### 3.1. Germinal Landscape of Nucleotide Variants in Proto-Oncogenes and Tumor Suppressor Genes

In a study conducted on a cohort of women clinically diagnosed with cervical cancer, 148 nucleotide sequence substitutions were identified in genes directly implicated in tumorigenesis (Table 2). Among them, 35 (23.6%) are classified as benign based on the literature and database information, while 105 (70.9%) are categorized as variants of uncertain significance (VUSs) whose clinical impact is yet to be determined. The majority of variants with uncertain significance (22 out of 26) were found in the *ROS1* gene, likely due to the inclusion of its intronic regions in the target panel.

Seven variants (4.7%) were classified as pathogenic or likely pathogenic. Among these, the rs1042522 polymorphic variant in the *TP53* gene has been associated with an increased risk of cervical cancer in several studies (0.6%) (Table 3).

### 3.2. Analysis of the Effect of P and LP Germinal Variants on the Course of Cervical Cancer

#### 3.2.1. Nucleotide Sequence Variants in the *TP53*Gene

Nucleotide substitutions in the *TP53* gene accounted for 4.7% of the detected variants among women with cervical cancer. Six out of seven variants belong to the category of variants of uncertain significance (VUS), and only one—the proline to arginine substitution at position 72—has been a topic of considerable scientific discussion.

In 87.0% of the patients (94/108), a missense substitution was identified in codon 72 of the *TP53* gene (c.215C > G, p.Pro72Arg, rs1042522, chr17:7579472), 44 of which were in the homozygous state, and 50 in the heterozygous state.

Mutations in the *TP53* gene are common genetic events in most types of cancer, leading to increased cell proliferation, inhibition of apoptosis, and often resulting in genetic instability. The effect of the p.Pro72Arg variant of the TP53 gene in different types of cancer is debated. For example, Storey A. and colleagues demonstrated that the Arg72 protein variant in the homozygous state increases susceptibility to HPV-associated cervical cancer sevenfold [15]. However, a subsequent study by Yasutaka Kawamata and colleagues showed that the expression level of the p53 protein in normal human keratinocytes for each codon 72 p53 genotype was not affected by the introduction of HPV 16-E6 from recombinant adenovirus, and *p53* mRNA was activated independently of the *p53* genotypes when HPV 16-E6 was expressed. Statistical analysis did not reveal a link between *TP53* genotypes and the development of cervical neoplasms [16]. Hugo Sousa and colleagues concluded, after reviewing the literature data and conducting meta-analyses, that this variant segregates differently among various ethnic groups. Therefore, the possible role of this genetic variant may be linked to a specific genetic background, which could explain why some studies report an increased risk of cervical cancer associated with the arginine variant of *TP53* [17]. Orsted D.D. and colleagues demonstrated that the p.Pro72Arg polymorphism affects overall survival in cancer patients in the general population. The 12-year overall survival rate increased by 3% (*p* = 0.003) in heterozygous p53Arg/Pro carriers and by 6% (*p* = 0.002) in p53Pro/Pro homozygotes compared to p53Arg/Arg homozygotes, which corresponds to a 3-year increase in median survival for Pro/Pro homozygotes compared to Arg/Arg. Thus, the *p53* Arg72Pro polymorphism leads to increased overall survival but does not reduce cancer risk [18]. In a later study involving a cohort of cervical cancer patients, the polymorphism was shown to affect overall survival, with p53Arg/Pro carriers having a median survival of 126 months compared to 111 months for p53Arg/Arg and p53Pro/Pro genotypes (*p* = 0.047) [19].

In our study, no significant associations were identified between the alleles and genotypes of the rs1042522 locus of the *TP53* gene and cervical cancer.

In 97% of the women, cervical cancer was diagnosed in late 2020 or early 2021, limiting our analysis to three-year survival data. Combined chemoradiotherapy was successful in 71 out of 106 patients (67%), while 35 (33%) did not achieve a sufficient therapeutic response; however, no correlations were found between genotypes and response to therapy. The median age of cervical cancer onset was 53.5 years for patients with the CC genotype, 52.3 years for CG, and 47.6 years for GG. This difference of up to 5.9 years in median age suggests an earlier onset of cervical cancer in carriers of the homozygous p53Arg/Arg variant.

The obtained results suggest that a pharmacogenomic approach based on patient genetic profiles, including *TP53* genotype analysis, could be used to individualize treatment and optimize therapeutic methods, potentially improving clinical outcomes and reducing toxicity.

#### 3.2.2. Co-Carriage of Variants in the *APC* and *BRAF* Genes in a Patient with Cervical Cancer

The p.(Ser836Ter) variant in the *APC* gene (c.2507C > G, chr5:112173798) in the heterozygous state was detected in one woman (0.92%). This substitution is considered pathogenic as it creates a stop codon and is predicted to result in premature truncation of the protein. According to the Uniprot database, mutations in this gene are associated with diseases such as familial adenomatous polyposis (FAP) type 1, hereditary desmoid disease, medulloblastoma, gastric cancer, hepatocellular carcinoma, gastric adenocarcinoma, and proximal gastric polyposis.

Germline variants in kinases identified in the study represent 45.3% of the detected variants (67 out of 148). Among these, only one variant in the *BRAF* gene is considered pathogenic.

The missense variant p.(Trp531Leu) in the *BRAF* gene (c.1592G > T, chr7:140476814, rs397507478) in the heterozygous state was detected in one woman (0.92%). According to the ClinVar database, this substitution is considered pathogenic; however, detailed functional analysis data for this variant are not available.Using the wild-type *BRAF* protein structure model built with ‘SWISS-MODEL’ and analyzed with ‘DynaMut2’, we determined that the substitution of tryptophan with leucine at position 531 results in destabilization of the protein, with a score of −2.42 kcal/mol (ΔΔGStability). Nevertheless, the substitution of codon TGG with TTG is predicted to be benign according to six prediction programs (SIFT, PolyPhen-2, CADD, REVEL, MetaLR, MutationAssessor).

However, given that, the p.Trp531Leu substitution occurs in the protein kinase domain and that a pathogenic variant leading to a nonsense codon beinglocated at the same genomic position, functional studies are required to accurately determine its clinical significance. The identification of activating mutations in the *BRAF* gene across various malignancies has facilitated the development of effective *BRAF* inhibitors, such as vemurafenib (Zelboraf) and dabrafenib [20]. Although the V600E mutation is most frequently identified among *BRAF* mutations, the spectrum of mutations in the *BRAF* gene continues to expand with the advent of next-generation sequencing [21].

Two missense substitutions, p.Ser836Ter in the *APC* gene and p.Trp531Leu in the *BRAF* gene, were identified in a patient born in 1959. She sought medical help in 2021 due to a recurrence of cervical cancer. The initial diagnosis was made in 2020 and was described as stage 4, grade 4 cervical cancer with metastases to the iliac lymph nodes and peritoneal metastases (T4N1M1) (according to ICD-10: C53.8). The patient passed away in November 2021, as the disease progressed aggressively. Such co-carriage of two mutations in the tumor suppressor gene and the serine/threonine protein kinase gene has not been described in the literature.

#### 3.2.3. Nucleotide Variants in the *CHEK2*Gene

Germline mutations in genes encoding proteins that regulate DNA repair and the response to double-strand DNA breaks have been recognized as pathogenic factors contributing to hereditary cancer predisposition.The *ATM-CHEK2-TP53* gene group has been shown to initiate the primary DNA damage response and acts as a barrier to cancer development. Pathogenic germline mutations in the *CHEK2* gene are among the most frequent alterations in various tumors, and their role has been confirmed in gender-specific cancers such as breast and prostate cancer [22].

Among the identified variants, substitutions in the *CHEK2* gene account for 6 out of 148 (4%) alterations. Five of these are categorized as VUSs, and one is pathogenic.

The missense variant p.(Ile157Thr) in the *CHEK2* gene (c.470T > C, rs17879961, chr22:29121087) in the heterozygous state was detected in three women (2.7%). One patient had a combination of cervical adenocarcinoma (T3bN0M0) and breast cancer. Cervical cancer manifested at the age of 73, with a fatal outcome within a year. In patients with squamous-cell cervical cancer, the disease manifested at ages 49 (T2bN1M0) and 62 (T2aN0M0). Although six in silico predictive programs interpret this substitution as benign (SIFT, PolyPhen-2, CADD, REVEL, MetaLR, MutationAssessor), it is still considered likely pathogenic in breast cancer [23] and pathogenic or likely pathogenic in cancer predisposition syndromes [24,25]. According to the ‘OncoBRCA’ database, this variant is classified as pathogenic or likely pathogenic for several types of cancer, including breast cancer and other malignancies. According to the ‘GnomAD’ database (v4.0.0.), the minor allele frequency of this variant is 0.003 in the general population. According to the ‘UniProt’ database, the substitution of isoleucine with threonine at position 157 of the *CHEK2* protein occurs in the FHA domain, a region critical for the protein’s role in DNA damage response. Using the wild-type protein structure built with ‘SWISS-MODEL’ and analyzed with ‘DynaMut2’, we found that this substitution is destabilizing, with a score of −1.2 kcal/mol (ΔΔGStability). As an additional study, we investigated the frequency of this substitution in a comparison group of healthy women with a history of spontaneous HPV elimination. Based on these findings, we concluded that these women are not predisposed to persistent HPV carriage and, consequently, to cervical cancer. As a result, we found that 3 (5.88%) of 51 women in the comparison group carried the pathogenic p.(Ile157Thr) variant in the *CHEK2* gene in the heterozygous state. Given that the frequency of this pathogenic variant is higher in the comparison group (5.88%) than in the cervical cancer patient group (2.7%), we concluded that this mutation is not associated with an increased risk of cervical cancer. However, it is still premature to draw conclusions about whether this substitution affects disease progression.

#### 3.2.4. Germinal Variants in the *BRCA2* Gene

BRCA1 and BRCA2 proteins play a key role in regulating DNA repair and maintaining genome integrity [26]. In our study, 16 (10.81%) variants were identified in these genes, one of which was pathogenic.

The missense variant p.(Arg3052Trp) in the *BRCA2* gene (c.9154C > T, chr13:32954180, rs45580035) in the heterozygous state was detected in onewoman (0.92%). According to multiple databases, this variant is classified as pathogenic for cancer predisposition syndromes, including hereditary breast and ovarian cancers. This variant was identified in a patient who developed squamous-cell cervical cancer (T3N1M0) at the age of 58, resulting in a fatal outcome within 2 years.

#### 3.2.5. Mutations in Genes of the Mismatch Repair System

Mutations in mismatch repair (*MMR*) genes, such as *MLH1*, *MSH2*, *MSH6*, and *PMS2*, are associated with hereditary nonpolyposis colorectal cancer (Lynch syndrome). The MMR system recognizes and corrects DNA errors caused by mismatched nucleotides. MSH proteins play a crucial role in recognizing these mismatches and initiating the repair process [27]. Seventeen out of 148 variants (11.4%) identified in our study were found in these genes. Of these, 3 out of 17 variants were pathogenic or likely pathogenic.

The variant p.(Trp117Ter) in the *MSH2* gene (c.350G > A, chr2:47635679, rs786202083) in the heterozygous state was detected in one woman (0.92%). This variant is a nonsense variant, resulting in the replacement of tryptophan with a premature stop codon (TGG > TAG). It is expected to result in the loss of normal protein function due to truncation or nonsense-mediated mRNA decay. This mutation was found in a 48-year-old woman with a combination of cervical cancer and endometrial cancer. Immunohistochemical analysis of her endometrial tumor revealed microsatellite instability, and she is currently receiving the ninthline of immunotherapy with a satisfactory therapeutic response.

**Figure 1:**
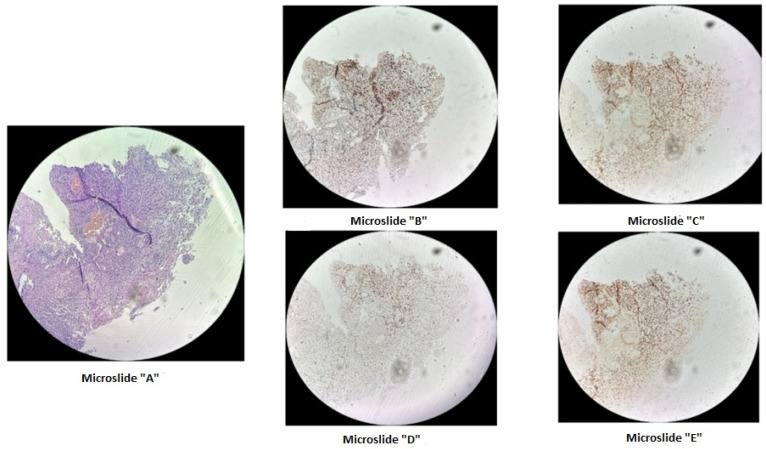
Immunohistochemical staining of cervical cancer preparation using the dMMR panel. Microslide (**A**): G-E staining at ×10 magnification; Microslide (**B**): PMS2 antibody staining at ×10 magnification; Microslide (**C**): MSH2 antibody staining at ×10 magnification; Microslide (**D**): MLH1 antibody staining at ×10 magnification; Microslide (**E**): MSH6 antibody staining at ×10 magnification. (Note: since the tissue block was prepared in 2015, some staining defects were observed due to the prolonged storage period of the material for immunohistochemical study; however, despite some staining heterogeneity, the expression of DNA mismatch repair proteins is preserved in viable tumor cells).

**Figure 2:**
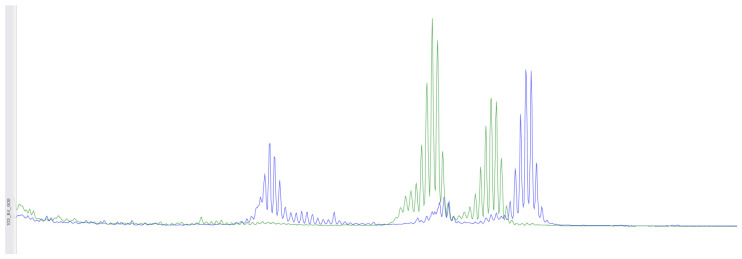
Using fragment analysis on a 3500xL genetic analyzer, the study investigated five microsatellite loci: NR-21, BAT-26, BAT-25, NR-24, NR-27. As a result of the study, no microsatellite instability was detected.

A microdeletion p.Asp706GlyfsTer11 - > G with a frameshift in the *MSH2* gene at genomic coordinate chr2:47703614 was found in a woman born in 1963 who was diagnosed with stage 3B, grade 2 squamous-cell cervical cancer in 2015, and at that time underwent a radical course of chemoradiotherapy (CRT). In 2021, due to disease recurrence, she underwent another course of CRT, followed by complications. The patient passed away in January 2022. This mutation is considered likely pathogenic, though its effects are not yet fully understood. However, reliable morphological and molecular genetic methods exist for diagnosing microsatellite instability in tissue. Therefore, we decided to conduct additional studies for this patient and others with similar mutations. On the diagnostic biopsy material of the primary tumor from a paraffin-embedded histological block from 2015, we performed an immunohistochemical study for microsatellite instability (dMMR) and did not detect loss of expression of MSH2, MSH6, MLH1, PMS2 in viable tumor cells (Figure 1). Additionally, using fragment analysis on a 3500xL genetic analyzer (Thermo Fisher Scientific, Waltham, MA, USA), we analyzed five microsatellite loci: NR-21, BAT26, BAT-25, NR-24, and NR-27 (Figure 2). The study revealed one allelic variation in the loci examined, indicating the absence of microsatellite instability. Previously, such results were interpreted as MSI-low; however, in 2018, at the ESMO consensus on the diagnosis of MMR deficiency, it was decided to consider the MSI-low status as non-existent [28]. Thus, we can conclude that the germline substitution p.Asp706GlyfsTer11, - > G did not have a pathogenic effect on the tumor and is not clinically significant.

**Figure 3:**
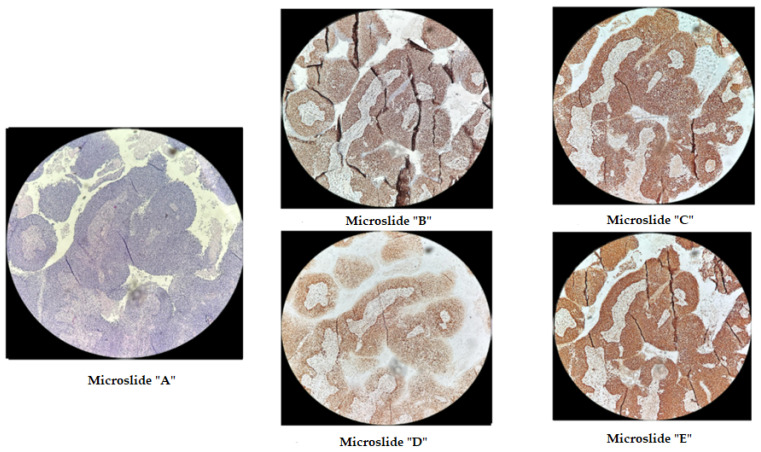
Immunohistochemical staining of cervical cancer preparation with the dMMR panel. Microslide (**A**): G-E staining, ×10; Microslide (**B**): PMS2 antibody staining, ×10; Microslide (**C**): MSH2 antibody staining, ×10; Microslide (**D**): MLH1 antibody staining, ×10; Microslide (**E**): MSH6 antibody staining, ×10 (note: preserved expression of mismatch repair proteins is observed).

**Figure 4:**
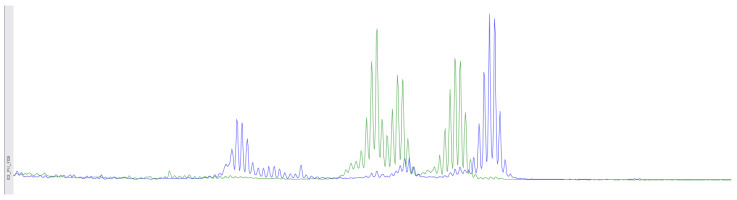
Using fragment analysis on a 3500xL genetic analyzer, the study investigated five microsatellite loci: NR-21, BAT-26, BAT-25, NR-24, NR-27. The study revealed allelic variations in the loci examined, indicating the presence of microsatellite instability (MSI-High).

The p.Phe1088LeufsTer5 frameshift variant in the *MSH6* gene at genomic coordinate chr2:48030639 was identified in a patient born in 1962 with moderately differentiated invasive squamous-cell cervical cancer (stage IIb T2bN1M1nod grade IV), which was diagnosed in November 2021. In February 2022, after the progression of the disease, she underwent further examination and received a course of chemoradiotherapy (CRT). The disease progressed in the summer of 2023, and the patient passed away in the autumn of 2023. Immunohistochemical analysis was performed on diagnostic biopsy material from the primary tumor in a paraffin-embedded histological block from 2022, demonstrating the retention of MSH2, MSH6, MLH1, and PMS2 protein expression in viable tumor cells (Figure 3). Additionally, fragment analysis on a 3500xL genetic analyzer investigated fivemicrosatellite loci: NR-21, BAT26, BAT-25, NR-24, NR-27. The study revealed twoallelic variants in the examined loci, indicating high microsatellite instability (MSI-High) (Figure 4). Thus, it can be concluded that the germline p.Phe1088LeufsTer5 variant is pathogenic and influenced the course of the disease. This case of microsatellite instability detection reveals contradictory results between the two MSI detection methods. The literature suggests that, in certain situations, the IHC test may produce false-negative results in the presence of certain missense mutations or *MLH1* promoter methylation [29]. It appears that in our case, the germline mutation in the *MSH6* gene, which was caused by a frameshift, should also be taken into consideration.

Based on the obtained results, it is advisable to use molecular genetic methods to detect microsatellite instability as a primary approach for patients with cervical cancer.

## 4. Conclusions

Cervical cancer (CC), like other malignancies, presents a complex and genetically heterogeneous disease profile. The clinical characteristics of the patients, along with the identified germline variants, enhance our understanding of the role of rare pathogenic and likely pathogenic mutations in the molecular pathogenesis of the disease. The clinical presentation of the patients suggests that pathogenic germline mutations significantly influence the course and outcome of the disease, leading to its deterioration.

From an oncogenetic perspective, these findings underscore the critical need tointegrate germline genetic screening into the routine diagnostic and therapeutic management of cervical cancer. The identification of pathogenic germline variants not only provides insights into the genetic predisposition and underlying molecular mechanisms driving the disease but also has profound implications for personalized treatment strategies. For instance, patients harboring certain mutations may benefit from targeted therapies, such as PARP inhibitors in the context of *BRCA* mutations, or immune checkpoint inhibitors in cases associated with microsatellite instability. Additionally, understanding the specific genetic alterations in CC can facilitate the development of novel therapeutic approaches aimed at mitigating the impact of these mutations.

Moreover, the identification of these germline mutations has implications beyond the affected individual, extending to at-risk family members who may also carry these variants. This highlights the importance of genetic counseling and testing as a critical component of oncogenetic care, enabling risk assessment and preventive measures for relatives.

Further research is warranted to delineate the full spectrum of germline mutations associated with cervical cancer and to clarify their functional roles in tumorigenesis. Large-scale genomic studies, coupled with functional assays, will be instrumental in uncovering novel genetic drivers and refining the classification of variants of uncertain significance (VUSs). This, in turn, may lead to the discovery of new biomarkers for early detection, prognosis, and therapeutic response, ultimately improving patient outcomes.

In conclusion, advancing our understanding of the genetic landscape of cervical cancer through comprehensive germline analysis represents a pivotal step toward more precise and effective cancer management, paving the way for a new era of personalized oncology.

## Figures and Tables

**Table 1:** Proto-oncogenes and Tumor Suppressor Genes Included in the NGS Target Panel (Gene Functions and Properties were obtained from the “NCBI” Database).

№	Genes	Function and Property	Analyzed Gene Regions
1	*RET*	A proto-oncogene that can undergo oncogenic activation through cytogenetic rearrangements and/or point mutations.	Exons 2, 5, 7, 9, 10, 12, 19
2	*MET*	A proto-oncogene that encodes the hepatocyte growth factor receptor (HGFR).	All codingregions
3	*KIT*	A proto-oncogene that encodes the receptor tyrosine kinase protein known as tyrosine-protein kinase KIT (CD117).	Exons 2, 3, 5, 8–20
4	*CHEK2*	The protein encoded by this gene is a regulator of cell cycle checkpoints and is considered a tumor suppressor. Moreover, this protein interacts with BRCA1 and phosphorylates it, facilitating BRCA1’s role in DNA repair after damage.	All codingregions
5	*ALK*	This gene encodes a receptor tyrosine kinase that belongs to the insulin receptor superfamily.	Exons 3–7, 9, 10, 12, 14–16, 20–28
6	*MSH2*	This locus is frequently mutated in hereditary nonpolyposis colorectal cancer. It is the human homolog of the mismatch repair gene.	All codingregions
7	*MSH6*	This gene encodes a member of the MutS family, responsible for mismatch repair.	Severalcriticalregions
8	*MLH1*	The protein encoded by this gene can heterodimerize with mismatch repair endonuclease PMS2, forming MutL alpha, a part of the DNA mismatch repair system.	All codingregions
9	*PIK3CA*	The protein encoded by this gene is a catalytic subunit that uses ATP for phosphorylating PtdIns, PtdIns4P, and PtdIns. This gene has been found to be oncogenic and involved in the development of cervical cancer.	Exons 2–6, 8, 10, 20, 21
10	*PDGFRA*	This gene encodes a cell surface receptor tyrosine kinase from the platelet-derived growth factor family, which is involved in cell signaling and tumor progression.	Exons 3–23
11	*TERT*	Encodes telomerase, a ribonucleoprotein polymerase that maintains the ends of telomeres by adding the telomeric repeat sequence TTAGGG. Aberrant regulation of telomerase expression in somatic cells may contribute to oncogenesis.	Promoter
12	*APC*	This gene encodes a tumor suppressor protein that inhibits the Wnt signaling pathway. It also participates in other processes, including cell migration and adhesion, transcriptional activation, and apoptosis.	Exons 2, 3, 5, 6, 7, 9, 10, 12–16
13	*ROS1*	This proto-oncogene is overexpressed in various tumor cell lines and belongs to the insulin receptor tyrosine kinase subfamily.	Exons 2, 10, 15, 16, 22, 23, 27, 37–42; Introns: 31, 32
14	*PMS2*	The protein encoded by this gene is a key component of the mismatch repair system, which functions to correct DNA mismatches, as well as small insertions and deletions that may arise during DNA replication and homologous recombination.	All codingregions
15	*EGFR*	The protein encoded by this gene is a transmembrane glycoprotein that is a member of the protein kinase superfamily. Mutationsinthisgeneareassociatedwithlungcancer.	All codingregions
16	*BRAF*	This gene encodes a protein that belongs to the RAF family of serine/threonine protein kinases. This protein plays a role in regulating the MAP kinase/ERK signaling pathway, which affects cell division, differentiation, and secretion. Mutations in this gene, particularly the V600E mutation, are frequently identified as cancer-causing in melanoma and are also found in various other cancers, including non-Hodgkin’s lymphoma, colorectal cancer, thyroid cancer, and non-small-cell lung cancer.	Exons 3, 8, 10–18
17	*CD274*	This gene encodes a ligand for the immunoinhibitory receptor (PD-L1), which is expressed on hematopoietic and non-hematopoietic cells such as T-cells and B-cells, as well as various tumor cell types.	All codingregions
18	*ABL1*	This gene represents a proto-oncogene that encodes a protein tyrosine kinase involved in various cellular processes, including cell division, adhesion, differentiation, and response to stress.	Exons 2, 4–8, 11
19	*BRCA2*	BRCA2 is involved in maintaining genome stability, particularly in the homologous recombination pathway for double-strand DNA repair.	All codingregions
20	*IDH2*	The protein encoded by this gene is a NADP(+)-dependent isocitrate dehydrogenase found in mitochondria. It plays a role in intermediary metabolism and energy production. This protein may be closely associated with or interact with the pyruvate dehydrogenase complex. Mutations in *IDH1* and *IDH2* are found in nearly 80% of oligodendrogliomas (GII and GIII), astrocytomas, and secondary glioblastomas.	Exon 4
21	*TP53*	This gene encodes a tumor suppressor protein that contains transcriptional activation, DNA binding, and oligomerization domains. The encoded protein responds to diverse cellular stresses to regulate the expression of target genes, thereby inducing cell cycle arrest, apoptosis, senescence, DNA repair, or changes in metabolism. Mutations in this gene are associated with various human cancers, including hereditary cancers such as Li–Fraumeni syndrome.	All codingregions
22	*ERBB2*	This gene encodes a member of the receptor tyrosine kinase family of epidermal growth factor receptors (EGFs). Amplification and/or overexpression of this gene has been reported in many cancers, including breast and ovarian tumors.	All codingregions
23	*BRCA1*	BRCA1 is involved in maintaining genome stability, particularly in the homologous recombination pathway for double-strand DNA repair.	All codingregions
No changes detected (n4)			
24	*IFNL3*	This gene encodes a cytokine distantly related to type I interferons and the IL-10 family. This gene, interleukin 28A (*IL28A*), and interleukin 29 (*IL29*) represent three closely related cytokine genes that form a cytokine gene cluster mapped to chromosome 19q13. Expression of the cytokines encoded by these three genes may be induced by viral infection. All three cytokines have been shown to interact with a heterodimeric class II cytokine receptor consisting of the interleukin 10 beta receptor (IL10RB) and the interleukin 28 alpha receptor (IL28RA). Since we are studying a tumor whose development is induced by a viral agent, mutations in such genes may be potentially targetable.	rs12979860 C > T
25	*PTEN*	This gene has been identified as a tumor suppressor, frequently mutated in a wide variety of cancers. The protein encoded by this gene is a phosphatidylinositol-3,4,5-trisphosphate 3-phosphatase.	All codingregions
26	*NRAS*	This N-ras oncogene encodes a membrane protein which shuttles between the Golgi apparatus and the plasma membrane. Mutations in this gene are associated with somatic colorectal cancer, follicular thyroid carcinoma, autoimmune lymphoproliferative syndrome, Noonan syndrome, and juvenile myelomonocytic leukemia.	Exons 2, 3, 4
27	*IDH1*	Isocitrate dehydrogenases catalyze the oxidative decarboxylation of isocitrate to 2-oxoglutarate. Mutations in *IDH1* and *IDH2* are found in nearly 80% of oligodendrogliomas (GII and GIII), astrocytomas, and secondary glioblastomas.	Exons 4, 8, 9

Gene information has been summarized from the NCBI Gene database (https://www.ncbi.nlm.nih.gov/gene) (accessed on 15 April 2024).

**Table 2:** Distribution of germline variants identified in women diagnosed with cervical cancer, categorized by genes and their associated signaling pathways.

Pathway	Genes	Total Numberof Mutations	P/LP	VUS
Cell Division Checkpoint	*APC*	11 (7.4%)	1	6
	*TP53*	7 (4.7%)	1	6
	*CHEK2*	6 (4.0%)	1	5
	*TERT*	1 (0.6%)	0	1
	*BRCA2*	11 (7.4%)	1	4
	*BRCA1*	5 (3.3%)	0	3
Regulators of Cell Proliferation, Migration, and Differentiation	*RET*	13 (8.7%)	0	7
	*MET*	11 (7.4%)	0	8
	*KIT*	7 (4.7%)	0	5
	*ALK*	7 (4.7%)	0	7
	*PIK3CA*	2 (1.3%)	0	1
	*PDGFRA*	3 (2.0%)	0	2
	*ROS1*	26 (17.5%)	0	26
	*EGFR*	8 (5.4%)	0	6
	*BRAF*	1 (0.6%)	1	0
	*ERBB2*	5 (3.3%)	0	3
	*ABL1*	5 (3.3%)	0	2
MicrosatelliteInstability (dMMR)	*MSH2*	3 (2.0%)	2	0
	*MSH6*	5 (3.3%)	1	4
	*MLH1*	2 (1.3%)	0	0
	*PMS2*	7 (4.7%)	0	7
Therapy Response Predictor	*IDH2*	1 (0.6%)	0	1
	*CD274*	1 (0.6%)	0	1

**Table 3:** Spectrum and frequencies of pathogenic and likely pathogenic variants in proto-oncogenes and tumor suppressor genes.

№	Variant	Position	Gene	Number/Frequency	Variant Significance	Described/Not Described
1	c.215G > C (p.Pro72Arg)	chr17:7579472	*TP53*	93 (86.1%)	Benign or likely pathogenic for GG variant	Described
2	c.470T > C (p.Ile157Thr)	chr22:29121087	*CHEK2*	3 (2.7%)	Pathogenic	Described
3	c.2507C > G (p.Ser836Ter)	chr5:112173798	*APC*	1 (0.92%)	Pathogenic	Described (not in this paper)
4	c.1592G > T (p.Trp531Leu)	chr7:140476814	*BRAF*	1 (0.92%)	Likelypathogenic	Described (not in this paper)
5	c.9154C > T (p.Arg3052Trp)	chr13:32954180	*BRCA2*	1 (0.92%)	Pathogenic	Described
6	c.350G > A (p.Trp117Ter)	chr2:47635679	*MSH2*	1 (0.92%)	Pathogenic	Described
7	- > G (p.Asp706GlyfsTer11)	chr2:47703614	*MSH2*	1 (0.92%)	Likelypathogenic	Not described
8	- > C (p.Phe1088LeufsTer5)	chr2:48030639	*MSH6*	1 (0.92%)	Likelypathogenic	Not described

## Data Availability

The data generated in the present study may be requested from the corresponding author.
